# The mediating role of coping style in the relationship between optimism and cognitive impairment in patients with stroke

**DOI:** 10.3389/fneur.2025.1674025

**Published:** 2026-01-12

**Authors:** Peiqi Gu, Limin Liu, Jie Su, Weifu Wang, Li Zhao

**Affiliations:** 1 School of Geriatrics and Elderly Care Industry, Shenyang Medical College, Shenyang, Liaoning, China; 2 The Second Affiliated Hospital of Shenyang Medical College, Shenyang, Liaoning, China; 3 Shenyang Red Cross Hospital, Shenyang, Liaoning, China; 4 School of International Education, Shenyang Medical College, Shenyang, Liaoning, China; 5 Department of Nursing, Beijing Health Vocational College, Beijing, China

**Keywords:** cognitive impairment, optimism, coping style, stroke, structural equation modeling

## Abstract

**Background:**

Stroke may bring psychological and cognitive challenges. Previous research revealed that several mediators, such as coping style and optimism, might be associated with cognitive impairment in patients with stroke.

**Objective:**

This study aims to establish a structural equation model of the relationships among optimism, coping style, and cognitive impairment, and to explore the mediating role of coping style in the association between optimism on cognitive impairment.

**Methods:**

Using a cross-sectional survey, 1,000 hospitalized patients with stroke from China were studied. The collected data were analyzed using correlation analyses, structural equation modeling, and regression analyses. SPSS 26.0 was used to construct logistic regression and decision tree models, and the area under the receiver operating characteristic (ROC) curve was used to evaluate the predictive performance of the two models. Structural equation modeling was used to examine the links among dispositional optimism, coping style, and cognitive impairment. The robustness of the model was verified using the bootstrap method.

**Results:**

The average scores of positive coping, negative coping, optimism, and cognitive impairment in patients with stroke were 19.26 ± 9.68, 10.49 ± 7.24, 23.22 ± 4.58, and 0.87 ± 1.69, respectively. The analysis of the receiver operating characteristic (ROC) curve showed that the predictive performance of logistic regression was slightly better than that of the decision tree model. SEM findings indicated that coping style serves as a significant mediator in the association between optimism and cognitive impairment.

**Conclusion:**

Both coping style and optimism were found to be significantly associated with cognitive impairment in patients with stroke, with coping style serving as a mediator of the association between optimism and cognitive impairment in this patient population. These cross-sectional findings suggest that, for inpatients with stroke, medical staff should consider paying attention to their cognitive function and assessing their psychological health status, such as their optimism and coping strategies, which may help inform the selection of supportive interventions that may benefit cognitive health.

## 1 Introduction

### 1.1 The burden of post-stroke cognitive impairment

Stroke is the second leading cause of death worldwide, with a very high disability rate. In China, there are over 3 million new cases of stroke every year (1). In recent years, with the improvement of medical technology, the survival rate and survival period of stroke patients have gradually been extended, but the longer survival period is also accompanied by sequelae that affect their quality of life (2). During the process of coping with the disease, stroke patients often experience negative emotions such as pessimism, disappointment, and low mood, which may be associated with poorer recovery and disease prognosis (3). Research shows that up to one-third of stroke patients will suffer from one or more social and psychological disorders after onset (4). Munthe mentioned in a review that the prevalence of mild cognitive impairment and severe cognitive impairment at 3 months after stroke is 14–29% and 11–42%, respectively (5). Overall, cognitive impairment shows a high incidence among stroke patients. Since stroke is an acute stress event that causes great physical and mental effects, it also indicates that, compared with neurodegenerative cognitive disorders, patients with post-stroke cognitive impairment may experience greater psychological pressure, depression, anxiety, and persistent psychological problems (6), which may potentially affect their subsequent rehabilitation process and quality of daily life.

### 1.2 The potential role of optimism and coping styles

According to Engel’s Biopsycho-Social Model (BPSM), proposed in 1977, biological and psychological factors play a significant role in the processes and outcomes of diseases (7). Given the severe psychological impact of stroke, patients often exhibit low levels of optimism. For stroke patients, the probability of experiencing negative emotions after a stroke is 20–60%, which may be related to an increase in disability and mortality rates (8). Coping style refers to the individual’s coping behaviors and cognitive activities toward stressful events, and different coping styles lead to different behavioral outcomes and have varying impacts on individuals (9). The stress response model proposed by Andreotti (10) suggests that excessive stress is associated with impaired neurocognitive function, and a person’s ability to cope with stress may influence the relationship between stress and neuropsychological outcomes, such as cognitive decline (11). In contrast, a meta-analysis (12) showed that, in addition to biological factors such as age, sex, and demographic characteristics, social relationship factors and neuropsychiatric symptoms are also influencing factors for cognitive impairment, and some positive psychological factors are associated with a lower risk of cognitive impairment. Scholars have pointed out that higher levels of optimism and better health behaviors are related to better health status, and these health behaviors may also be beneficial for cognitive function (13, 14). Therefore, optimism and coping style represent promising research foci in understanding and potentially mitigating cognitive impairment in stroke patients.

### 1.3 The rationale and novelty of the current investigation

**Figure 1:**
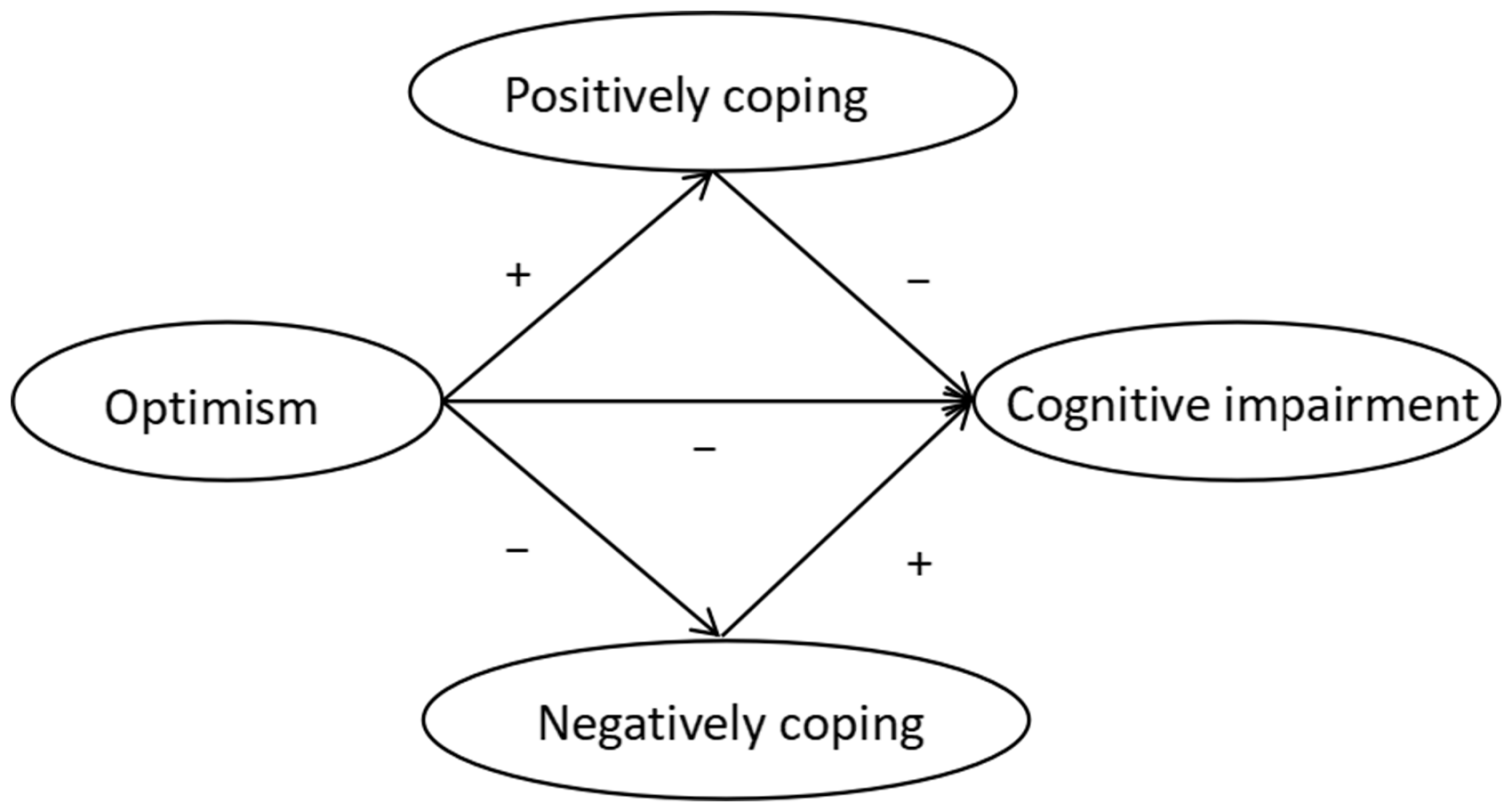
The study hypotheses.

The majority of existing studies examine the relationship between optimism or coping strategies and post-stroke cognitive function separately (15, 16). There is a lack of research on whether coping strategies mediate the relationship between optimism and cognitive impairment by integrating the three into an integrated model, particularly in stroke patients. This study aims to investigate the relationship between optimism, coping styles, and cognitive impairment. Additionally, we attempt to better understand the underlying psychological mechanisms of cognitive function in patients with stroke to provide insights for future prevention strategies. Based on previous theories and research, we propose study hypotheses as shown in Figure 1.

Hypotheses:

*H1:* Optimism is hypothesized to be positively associated with positive coping.

*H2:* Optimism is hypothesized to be negatively associated with negative coping.

*H3:* Positive coping is hypothesized to be negatively associated with cognitive impairment.

*H4:* Negative coping is hypothesized to be positively associated with cognitive impairment.

*H5:* Optimism is hypothesized to be negatively associated with cognitive impairment.

*H6:* Coping style is hypothesized to mediate the association between optimism and cognitive impairment.

## 2 Materials and methods

### 2.1 Research participants

This study used a stratified sampling method. First, we selected a representative comprehensive hospital from each district of Shenyang City, and these hospitals were all designated treatment hospitals on the “Emergency treatment for stroke in Shenyang” network. This sampling approach aimed to obtain a sample from established and standardized stroke care settings within the region. Second, within these hospitals, we recruited patients who met the inclusion criteria during the data collection period. A total of 1,000 valid questionnaires were collected, with an effective response rate of 92.5%. According to the Kendall criterion in statistics, the sample size should be at least 5–10 times the number of independent variables. This research questionnaire comprised a total of 36 items, and after screening, a sample of 1,000 participants was included. Inclusion criteria were as follows: (1) ≥18 years old; (2) patients diagnosed with stroke in clinical settings; (3) clear consciousness and stable condition; and (4) patients who provided informed consent and voluntarily participated in this study. All participants signed informed consent forms. The exclusion criteria were as follows: (1) patients with other serious acute diseases requiring strict bed rest; (2) patients with cognitive impairment, auditory dysfunction, or other conditions affecting the completion of the questionnaire; and (3) patients with a confirmed diagnosis of a mental disorder.

Investigators conducting this research were trained in appropriate content and questionnaire scoring methods, and the target and content of these questionnaires were described to eligible patients in a uniform manner. All patients provided informed consent prior to study participation. Questionnaires were distributed, and data were collected through one-on-one interviews and reviewed on the spot. Collected questionnaires were individually numbered, and a double-entry approach was employed to ensure that all data entry was accurate. The Ethics Committee of Shenyang Medical College approved the present research.

### 2.2 Research tools

#### 2.2.1 Demographic characteristics

Demographic data include sex, age, ethnicity, height, weight, literacy level, BMI, marital status, work situation, area of residence, family monthly income, chronic disease, first onset of stroke, passive smoking, smoking, alcohol consumption, daily exercise time, and type of stroke.

#### 2.2.2 The six-item life orientation test-revised (LOT-R)

LOT-R is a self-assessment scale used to measure optimistic personality (17). It consists of 10 items, 4 of which are filler items designed to mask the fundamental purpose of the test. Of the six rating items, three assess optimism and three assess pessimism. Respondents indicate their agreement with each item on a 5-point scale ranging from 0 (strongly disagree) to 4 (strongly agree). The total score is calculated by adding the raw scores of the optimistic items and the inverted raw scores of the pessimistic items. The score range is from zero to 24, and a higher score indicates a higher level of optimism, while a lower score indicates a lower level of optimism, commonly referred to as pessimism. This established measure demonstrates robust psychometric properties, such as good reliability, validity (both discriminant and convergent), and stable item performance. Previous research supports the temporal stability of LOT-R-assessed optimism (14). In the current study, the scale yielded a Cronbach’s *α* of 0.93.

#### 2.2.3 The simplified coping style questionnaire (SCSQ)

Coping styles were evaluated using the 20-item Simplified Coping Style Questionnaire (SCSQ) (18). This self-report instrument measures two dimensions: positive and negative coping. Respondents indicate how frequently they employ each strategy on a 4-point scale (1 = never to 4 = always). The total SCSQ score, calculated by subtracting the negative dimension standard score from the positive one, reflects an individual’s propensity toward positive coping (higher scores indicate greater use). Although the SCSQ was initially developed in a general population, it has been widely used and has established good psychometric properties in populations with stroke or other chronic diseases (19). In the current sample, Cronbach’s *α* was 0.93 overall, with subscale α values of 0.94 (positive coping) and 0.85 (negative coping).

#### 2.2.4 Ascertain dementia 8 (AD8)

The AD8 scale is a rapid screening tool developed by the University of Washington in the United States in 2005. It includes eight items: judgment, hobbies, repeating the same thing repeatedly, learning how to use tools, appliances, or small tools, forgetting the correct year and month, dealing with complex financial problems, remembering an agreed-upon time, and thinking and memorizing. Each item requires the test subject to answer “no change,” “changed,” or “do not know.” Answering “changed” earns 1 point, while answering “no change” or “do not know” earns 0 points. We consider an AD8 score of 2 or higher as a significant abnormality, indicating the presence of significant cognitive impairment. As a short and rapid questionnaire used for general screening, The AD8 is highly correlated with clinical neuroassessment (19) and other screening tools such as the Montreal Cognitive Assessment (20), and has been widely used in large-scale cohort studies of the general population and surgical patients (21). However, the optimal threshold for the AD8 may vary depending on different uses or age groups, but in various studies (22), AD8 scores ≥ 2 are more sensitive to early cognitive changes in the Asian population, especially Chinese populations (23, 24). In subsequent research, it would be more scientific to choose appropriate cutoff scores based on different populations. In addition, relevant studies have shown that serum biomarkers may also be associated with early cognitive changes in emergency patients (25), such as serum uric acid levels. Therefore, in subsequent studies, laboratory test indicators can be further combined with evaluation to more accurately screen patients with cognitive impairment.

### 2.3 Statistical analysis

The study was statistically analyzed using SPSS, with a two-tailed *p* < 0.05 indicating a statistically significant difference. *T*-test and one-way ANOVA were used to describe the distribution and significant differences of optimism, coping style, and cognitive impairment in cerebrovascular patients with different demographic characteristics. Spearman correlation analysis was used to assess the correlation between optimism, coping style, and cognitive impairment in cerebrovascular patients.

A decision tree is a machine learning algorithm that offers advantages such as visualization, ease of interpretation, and high accuracy. Studies have shown that decision trees can achieve higher predictive performance for disease progression compared to traditional logistic regression models. Therefore, this study will use logistic regression and a decision tree to construct a risk prediction model for cognitive impairment in stroke patients, and compare the predictive performance of the two, in order to guide clinical identification and prevention of high-risk patients with cognitive impairment. Structural equation model analysis was performed using AMOS, with optimism as the independent variable, cognitive impairment as the dependent variable, and coping style as the mediating variable; parameter estimation was performed using the maximum likelihood method. SEM was preferred over traditional regression-based mediation analysis: (a) it allows for the simultaneous estimation of all hypothesized paths within a single, comprehensive model, providing a more holistic view of the relationships; (b) it explicitly accounts for measurement error in the latent constructs, leading to more accurate parameter estimates; and (c) it provides goodness-of-fit indices to evaluate how well the proposed theoretical model reproduces the observed data, which regression methods cannot offer. Our mediation analysis aims to test the plausibility of a theoretical model and examine indirect associations based on established psychological theory, rather than to establish causal pathways. Previous studies have also shown that this method can elucidate the relationships between variables in cross-sectional studies (26, 27).

## 3 Results

### 3.1 Demographic characteristics

Screening for post-stroke cognitive impairment using the AD8 scale, based on established criteria and previous studies (28), we use a total score of ≥2 as the cutoff value to classify the presence of cognitive impairment. This method is particularly suitable for our study population, as the AD8 relies on informant reporting, and with fewer questions and shorter answer times, it is suitable for large-scale screening. A total of 1,000 patients with stroke completed the questionnaire survey, of which 180 patients (18%) had cognitive impairment, and their AD8 score was greater than or equal to 2 points. Moreover, monthly income, smoking, alcohol consumption, the first onset of stroke, and the type of stroke showed a statistically significant difference in positive coping scores (*p* < 0.05). Marital status, BMI, monthly income, diabetes, smoking, alcohol consumption, first onset of stroke, the type of stroke, and coronary heart disease showed a statistically significant difference in negative coping scores (*p* < 0.05). Age, literacy level, marital status, monthly income, home location, hypertension, diabetes, coronary heart disease, fatty liver, alcohol consumption, physical activity time, first onset of stroke, and type of stroke showed a statistically significant difference in optimism (*p* < 0.05). Age, BMI, marital status, home location, diabetes, fatty liver, alcohol consumption, smoking, physical activity time, and type of stroke showed a statistically significant difference in cognitive impairment (*p* < 0.05). The demographic characteristics of the subjects with and without cognitive impairment are summarized in Table 1.

**Table 1:** Demographic characteristics of stroke patients.

Characteristic	*N* (%)	Coping style		Optimism	Cognitive impairment
		Positive coping	Negative coping		
Sex					
Male	588 (58.8%)	18.68 ± 9.55	10.20 ± 7.12	23.15 ± 4.69	0.80 ± 1.69
Female	412 (41.2%)	19.84 ± 9.82	10.77 ± 7.49	23.22 ± 4.70	0.85 ± 1.77
Age					
<60	178 (17.8%)	21.10 ± 9.76	10.85 ± 7.63	24.47 ± 4.23	0.35 ± 1.00
≥60	822 (82.2%)	18.78 ± 9.61	10.36 ± 7.21	22.94 ± 4.74**	0.90 ± 1.80*
Ethnic group					
Han Chinese	962 (96.2%)	19.21 ± 9.70	10.45 ± 7.29	23.20 ± 4.71	0.83 ± 1.72
Others	38 (3.8%)	17.82 ± 9.07	10.08 ± 7.11	22.76 ± 4.06	0.58 ± 1.68
BMI					
<24.0	458 (45.8%)	20.37 ± 9.49	11.66 ± 7.48	23.79 ± 4.61	0.72 ± 1.63*
≥24.0	542 (54.2%)	18.12 ± 9.71	9.38 ± 6.93**	22.66 ± 4.70	0.90 ± 1.79
Literacy level					
High school and below	804 (80.4%)	18.73 ± 9.72	10.45 ± 7.19	22.93 ± 4.73	0.84 ± 1.76
Above high school	196 (19.6%)	20.88 ± 9.27	10.36 ± 7.63	24.19 ± 4.36**	0.71 ± 1.56
Marital status					
Married	880 (88.0%)	19.29 ± 9.73	10.56 ± 7.34	23.31 ± 4.62	0.75 ± 1.63
Others	120 (12.0%)	18.12 ± 9.20	9.48 ± 6.70*	22.24 ± 5.05*	1.31 ± 2.23**
Work situation					
Retirement	800 (80.0%)	18.95 ± 9.70	10.45 ± 7.23	23.00 ± 4.73	0.86 ± 1.77
Others	200 (20.0%)	19.97 ± 9.55	10.36 ± 7.46	23.90 ± 4.44	0.64 ± 1.52
Monthly income					
≤4,000	560 (56.0%)	17.30 ± 9.75	9.81 ± 7.05	22.48 ± 4.85	0.86 ± 1.81
>4,000	440 (44.0%)	21.51 ± 9.04**	11.22 ± 7.48**	24.06 ± 4.32**	0.77 ± 1.60
Home location					
Rural	198 (19.8%)	21.26 ± 9.70	13.27 ± 6.94	23.20 ± 5.43	1.06 ± 1.76
Urban	802 (80.2%)	18.63 ± 9.60	9.73 ± 7.19	23.17 ± 4.49**	0.76 ± 1.71*
Hypertension					
No	387 (38.7%)	19.39 ± 9.53	10.49 ± 7.26	23.37 ± 4.48	0.78 ± 1.58
Yes	613 (61.3%)	19.00 ± 9.76	10.39 ± 7.29	23.06 ± 4.81*	0.84 ± 1.80
Diabetes					
No	770 (77.0%)	20.03 ± 9.68	10.93 ± 7.45	23.54 ± 4.55	0.71 ± 1.59
Yes	230 (23.0%)	16.21 ± 9.06	8.73 ± 6.38**	21.97 ± 4.95*	1.18 ± 2.06**
Has thrombolysis been administered					
No	856 (85.6%)	19.76 ± 9.64	10.64 ± 7.44	23.46 ± 4.56	0.73 ± 1.59
Yes	144 (14.4%)	15.57 ± 9.07	9.14 ± 6.03**	21.47 ± 5.06**	1.36 ± 2.27
Fatty liver					
No	940 (94.0%)	19.39 ± 9.68	10.42 ± 7.32	23.37 ± 4.56	0.71 ± 1.58
Yes	60 (6.0%)	15.46 ± 8.78	10.55 ± 6.53	20.18 ± 5.64**	2.45 ± 2.78**
Smoking status					
No	718 (71.8%)	20.02 ± 9.81	10.78 ± 7.50	23.39 ± 4.67	0.76 ± 1.71
Yes	282 (28.2%)	16.94 ± 8.94*	9.52 ± 6.58**	22.63 ± 4.69	0.96 ± 1.75**
Passive smoking					
No	715 (71.5%)	19.64 ± 9.61	10.52 ± 7.37	23.43 ± 4.68	0.84 ± 1.79
Yes	285 (28.5%)	17.94 ± 9.73	10.19 ± 7.04	22.54 ± 4.65	0.76 ± 1.54
Alcohol consumption					
No	737 (73.7%)	20.56 ± 9.78	11.23 ± 7.56	23.83 ± 4.46	0.62 ± 1.49
Yes	263 (26.3%)	15.21 ± 8.16**	8.19 ± 5.85**	21.34 ± 4.83*	1.36 ± 2.15**
Physical activity time					
≤1 h	442 (44.2%)	17.27 ± 9.42	10.17 ± 7.18	22.20 ± 5.05	1.24 ± 2.11
>1 h	558 (55.8%)	20.65 ± 9.62	10.63 ± 7.35	23.95 ± 4.23**	0.48 ± 1.23**
First onset of stroke					
No	404 (40.4%)	16.47 ± 8.86	9.31 ± 6.70	22.18 ± 4.86	0.98 ± 2.01
Yes	596 (59.6%)	20.97 ± 9.78**	11.18 ± 7.56**	23.85 ± 4.45**	0.70 ± 1.49
Type of stroke					
Ischemic stroke	752 (75.2%)	20.04 ± 9.96	8.00 ± 6.03	23.59 ± 4.60	0.76 ± 1.70*
TIA	248 (24.8%)	20.56 ± 8.33*	7.96 ± 5.65**	24.47 ± 5.33*	0.56 ± 1.74

*p < 0.05, **p < 0.01.

### 3.2 Correlation between optimism, coping style, and cognitive impairment

Spearman correlation analysis was used to assess optimism, positive coping, negative coping, and cognitive impairment scales. The results show that optimism was significantly positively correlated with positive coping (*r* = 0.684, *p* < 0.01), optimism was significantly negatively correlated with negative coping (*r* = −0.434, *p* < 0.01), and positive coping was significantly negatively correlated with cognitive impairment (*r* = −0.193, *p* < 0.01; Table 2).

**Table 2:** Correlation between optimism, coping style, and cognitive impairment.

Variables	Optimism	Positive coping	Negative coping	Cognitive impairment
Optimism	1			
Positive coping	0.684**	1		
Negative coping	0.434**	0.670**	1	
Cognitive impairment	−0.363**	−0.193**	−0.005	1

**p < 0.01.

### 3.3 Logistic regression analysis of factors influencing cognitive impairment in stroke patients

Using cognitive impairment in stroke patients as the dependent variable (0 = no, 1 = yes), binary logistic regression analysis was conducted with 10 statistically significant factors identified in univariate analysis as independent variables. The results showed that marital status, age, family location, alcohol consumption, exercise time, diabetes, and type of stroke were the main factors influencing cognitive impairment in elderly stroke patients. Among them, marital status, alcohol consumption, smoking, and ischemic stroke type were risk factors for cognitive impairment, while residing in a city, exercising for more than 1 h per day, and being under 60 years of age were protective factors, as shown in Table 3.

**Table 3:** Logistic regression analysis of factors influencing cognitive impairment in stroke patients.

Variable	Classification	*B*	SE	*Wald*	*p*	OR	95%*CI*
Marital status	Married	0.606	0.267	5.149	<0.05	1.833	(1.086, 3.093)
	Other						
Age	<60	0.745	0.272	7.531	<0.01	2.170	(1.237, 3.587)
	≥60						
BMI	<24.0	−0.020	0.024	0.678	0.410	0.980	(0.935, 1.028)
	≥24.0						
Home location	Rural	−0.667	0.235	8.017	<0.01	0.513	(0.324, 0.815)
	City						
Smoking status	No	0.032	0.226	0.02	0.888	1.032	(0.663, 1.607)
	Yes						
Alcohol consumption	No	0.763	0.218	12.196	<0.01	2.145	(1.398, 3.291)
	Yes						
Physical activity time	≤1 h/day	−0.868	0.202	18.467	<0.01	0.420	(0.283, 0.624)
	>1 h/day						
Suffering from diabetes	No	0.325	0.225	2.091	0.148	1.384	(0.891, 2.151)
	Yes						
Suffering from fatty liver	No (control)	0.76	0.363	4.388	<0.05	2.137	(1.050, 4.350)
	Yes						
Types of stroke	TIA (control)	−0.98	0.220	19.883	<0.01	0.375	(0.244, 0.577)
	Ischemic stroke						

### 3.4 Decision tree model score of influencing factors of cognitive impairment in stroke patients

**Figure 2:**
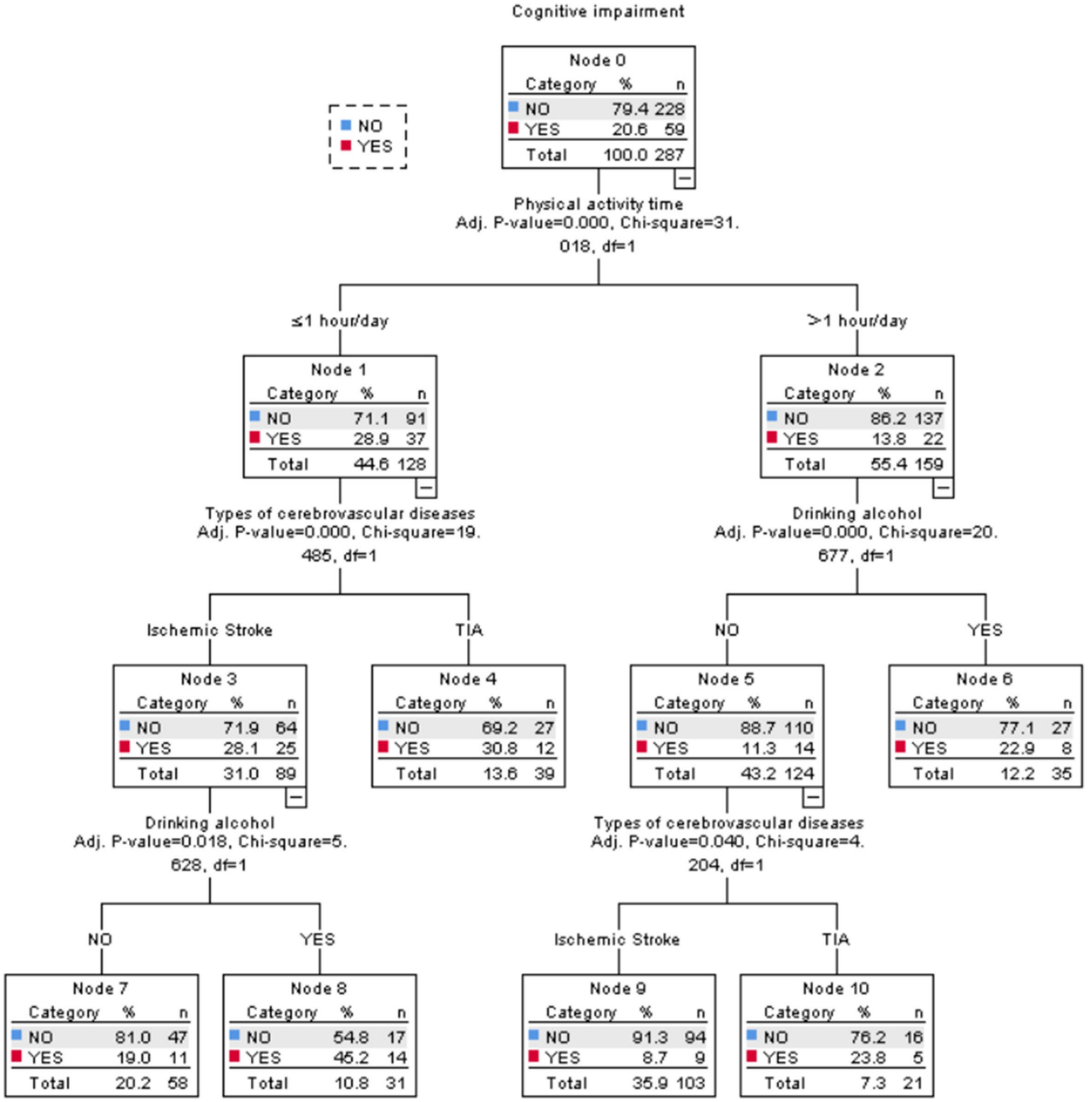
Decision tree model of cognitive impairment in elderly stroke patients.

Ten independent variables with statistical significance in univariate analysis, such as age, BMI, marital status, home location, smoking, alcohol consumption, exercise time, diabetes, fatty liver, and stroke type, were included in the decision tree model, as shown in Figure 2. The decision tree model has four layers and 11 nodes in total, including six terminal nodes. Three explanatory variables are screened out, such as alcohol consumption, exercise time, and the type of stroke. The daily exercise time is an important predictor of cognitive impairment in stroke patients.

### 3.5 Comparison of the prediction effect between logistic regression and the decision tree model

**Figure 3:**
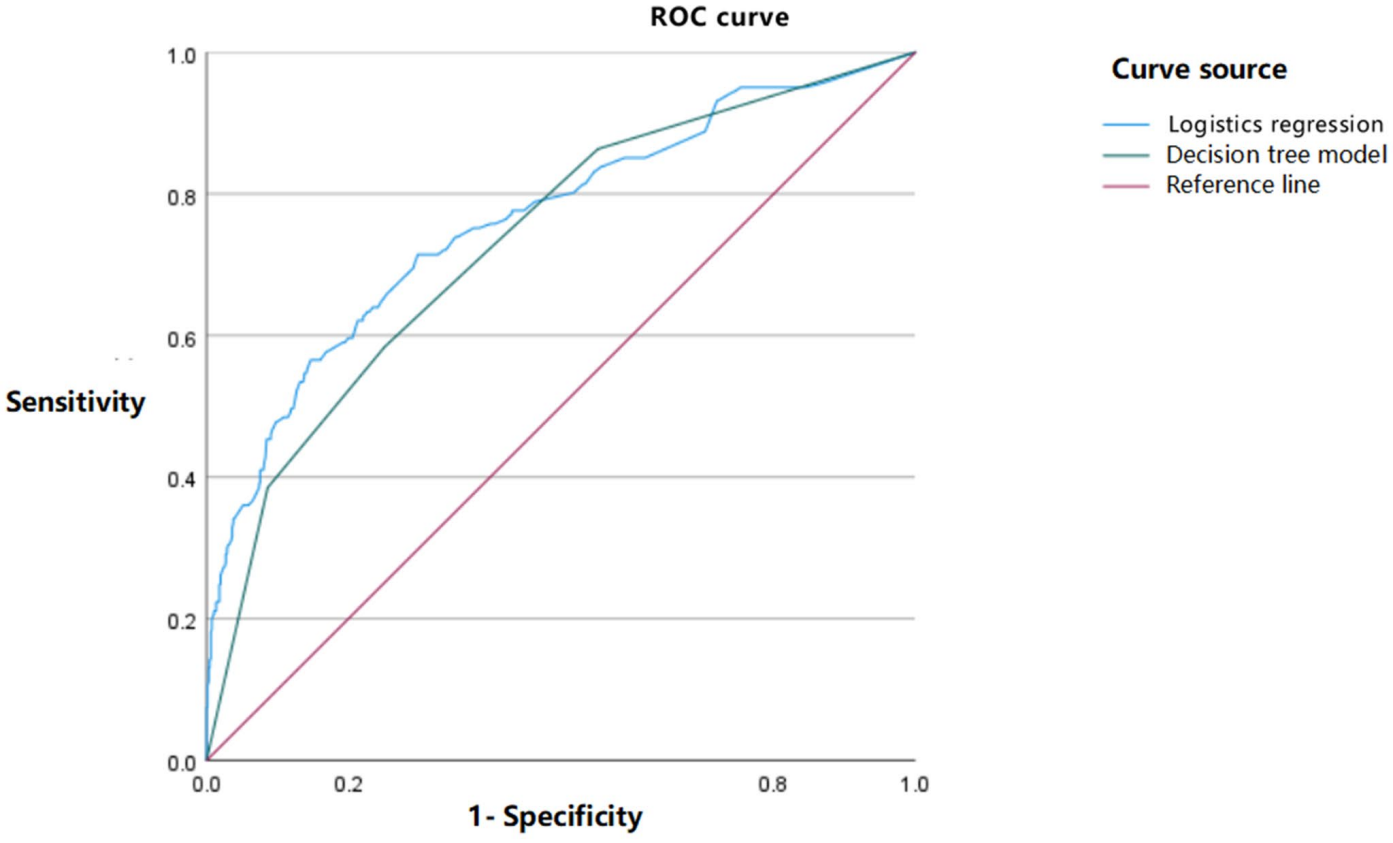
ROC curve of logistic regression and decision tree model.

By drawing the receiver operating characteristic (ROC) curve, the predictive effect of logistic regression and decision tree models on cognitive impairment in elderly stroke patients was analyzed. The results showed that the sensitivity of logistic regression (56.5%) was lower than that of the decision tree model (58.4%), and the specificity (85.3%) was higher than that of the decision tree model (74.9%). The area under the ROC curve (AUC) value of logistic regression was 0.762 (95% CI: 0.717–0.807), while the AUC value of the decision tree model was 0.732 (95% CI: 0.717–0.807). The prediction effect of logistic regression was slightly better than that of the decision tree model, as shown in Figure 3.

### 3.6 Factors influencing cognitive impairment in stroke patients

**Figure 4:**
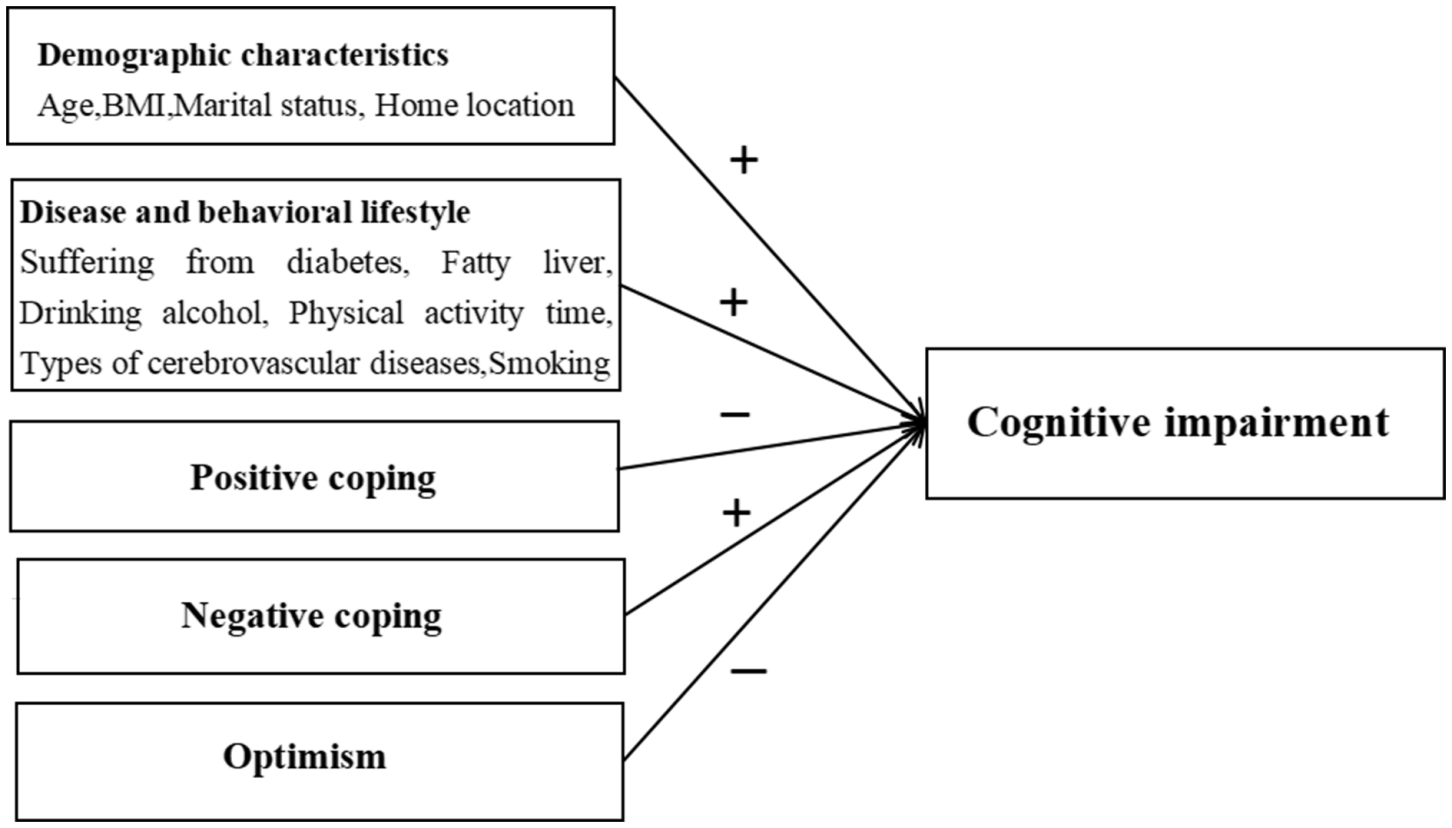
Factors influencing cognitive impairment in patients with stroke.

All variables related to cognitive impairment in the above statistical analysis were summarized to obtain demographic characteristics in Figure 4. There is a statistically significant difference (*p* < 0.05) in the impact of age, BMI, marital status, and family location on cognitive impairment. Factors related to disease and lifestyle behavior, such as diabetes, fatty liver, alcohol consumption, time of physical activity, and type of stroke, have statistically significant effects on cognitive impairment (*p* < 0.05), while positive coping has a negative impact on cognitive impairment, and negative coping has a positive impact on cognitive impairment. This means that stroke patients who adopt negative coping strategies may have lower cognitive function than those who adopt negative coping strategies. Meanwhile, more optimistic stroke patients may have better cognitive abilities.

### 3.7 Structural equation modeling analysis of optimism, coping style, and cognitive impairment

A structural equation model was constructed using AMOS 24.0, with optimism of patients with stroke as the independent variable, positive and negative coping as mediating variables, and cognitive impairment as the dependent variable. The measurement model was assessed to confirm the adequacy of the latent variable structures. Standardized regression weights for all indicators are presented in Table 4, all of which were statistically significant and exceeded the recommended threshold of 0.50, supporting convergent validity.

**Table 4:** Standardized regression weights for all indicators.

Latent variable	Observation indicators/sub-scales	Standardized regression weights	Standard error (S.E.)	*p*-value
Optimism	Item 1	0.57	0.078	<0.01
	Item 2	0.85	0.095	<0.01
	Item 3	0.82	0.095	<0.01
	Item 4	0.64	0.065	<0.01
	Item 5	0.83	0.089	<0.01
	Item 6	0.62	0.060	<0.01
Coping style	Positive coping	0.84	0.029	<0.01
	Negative coping	0.71	0.041	<0.01
AD8	Item 1	0.52	0.098	<0.01
	Item 2	0.68	0.090	<0.01
	Item 3	0.71	0.092	<0.01
	Item 4	0.69	0.089	<0.01
	Item 5	0.60	0.080	<0.01
	Item 6	0.70	0.099	<0.01
	Item 7	0.72	0.095	<0.01
	Item 8	0.65	0.094	<0.01

**Figure 5:**
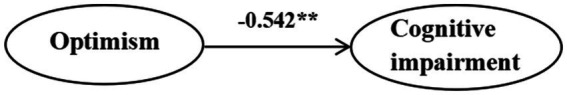
Direct effect of optimism and cognitive impairment in stroke patients.

**Figure 6:**
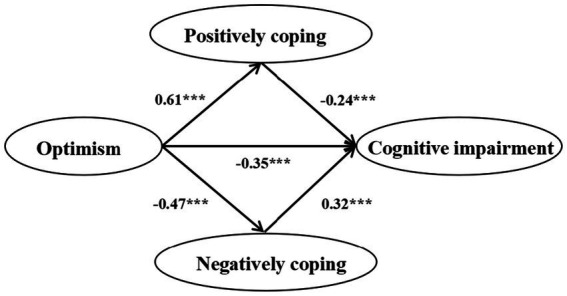
Structural equation modeling of coping style in optimism and cognitive impairment in cerebrovascular patients.

The model results indicate that optimism not only directly affects cognitive impairment but also has a significant indirect impact on cognitive impairment through coping strategies. The direct pathway of optimism affecting cognitive impairment is shown in Figure 5, and its goodness of fit is shown in Table 5, indicating that optimism has a significant impact on cognitive impairment (*β* = −0.542, *p* < 0.01). The indirect pathway from optimism to cognitive impairment mediated by coping strategies is shown in Figure 6, with goodness of fit *χ*^2^/df < 5, *p* < 0.05, CFI = 0.942, NFI = 0.925, IFI = 0.942, TLI = 0.905, RMSEA = 0.064. The results showed that positive coping had a negative effect on cognitive impairment (*β* = −0.24, *p* < 0.001), while negative coping had a positive effect on cognitive impairment (*β* = 0.32, *p* < 0.001). The path coefficient of optimism on cognitive impairment decreases with the mediating effect of coping strategies (*β* = −0.35, *p* < 0.001).

**Table 5:** Structural equation model fitting index.

Indicators	*χ*^2^/df	CFI	RMSEA	TLI	IFI	NFI	SRMR
Standard value	<5	>0.9	<0.10	<0.05	>0.9	>0.9	<0.05
Price	4.118	0.942	0.064	0.905	0.942	0.925	0.038

## 4 Discussion

### 4.1 Incidence of cognitive impairment in stroke patients

The incidence of PSCI in this study was 18%, slightly lower than the previously reported incidence of 20–40% in a meta-analysis and cohort studies (29, 30). This difference may be due to the following reasons. First, as this study mainly investigated stroke patients in the hospital, hospitalized patients generally have a shorter course of illness. However, studies have shown (31) that with the passage of time and the long-term damage to cerebral blood vessels caused by stroke, cognitive impairment symptoms only appear a few months after the occurrence of stroke. This may be one of the reasons why the results of this study are lower than those of other studies. In addition, the sample of this study came from designated stroke treatment hospitals in Shenyang, which have higher medical standards for stroke and may have prevented cognitive impairment to some extent. Furthermore, the differences in the number of selected samples and regions are also common reasons for the differences in research results.

### 4.2 Influencing factors of cognitive impairment in stroke patients

The results of this study showed that demographic variables, such as age, BMI, marital status, and family location, and factors related to disease and behavior lifestyle, such as diabetes, fatty liver, alcohol consumption, time of physical activity, smoking, and stroke type, have statistically significant effects on cognitive impairment, while positive coping was negatively correlated with cognitive impairment, and negative coping was positively correlated with it. Moreover, older age was linked to more severe cognitive impairment, which is closely related to neurobiological changes, brain structure degradation, lifestyle and environmental factors, and psychological and social factors (32). Individuals with a higher BMI also exhibit poorer cognitive ability, consistent with the findings of Beeri (33), which showed that higher BMI variability is associated with a faster decline in cognitive function. This may be related to progressive homeostasis imbalance in stroke patients with abnormal BMI, promoting harmful processes that lead to anoxic cell dysfunction, such as neuronal death, nerve degeneration, and other related factors. The degree of cognitive impairment of stroke patients is still different between urban and rural areas. The degree of cognitive impairment of stroke patients in rural areas is higher. The reason may be due to the lack of social structure resources in rural areas (34). In addition, stroke patients with a per capita monthly income of less than 4,000 yuan in this study have a higher probability of cognitive impairment, which has been demonstrated in previous articles (35). The reason is that the cognitive burden caused by poverty is equivalent to losing a whole night’s sleep. This kind of mental pressure will reduce the individual’s cognitive performance and may aggravate wrong decision-making and financial choices, so that the individual will fall into a worse psychological state. Depression in patients with physical activity for more than 1 h was less likely to be associated with cognitive impairment symptoms, which may be because exercise increases the release of some neurotransmitters, improves negative emotions, and reduces cognitive impairment symptoms (36). Previous studies have shown that alcohol consumption is a risk factor for cognitive impairment, which is consistent with the results of this study. Alcohol consumption was identified as a risk factor. These studies have pointed out that long-term heavy alcohol consumption will lead to changes in brain structure and function, such as hippocampal atrophy and white matter fiber damage, which are closely related to the decline of cognitive ability (37).

### 4.3 Predictive performance analysis

A decision tree is a machine learning method based on tree structure, mainly used for classification and regression tasks, capable of handling non-linear relationships (38). The decision tree model constructed in this study consists of four layers, 11 nodes, and six endpoints. Three explanatory variables were selected: alcohol consumption, daily exercise duration, and stroke type were jointly predicted by the two models. The difference in analysis between the two models is due to the different testing methods (39). The logistic regression model can accurately determine the linear relationship between independent and dependent variables by outputting OR values, while the decision tree model can display the interaction between different variables, gradually reduce the sample size during the analysis process, and screen for variables with more clinical significance. The results are expressed in the form of images, which is convenient for clinical application. However, using a single method may have limitations. Combining a logistic regression model with a decision tree model analysis is expected to provide a more comprehensive and accurate analysis of the influencing factors of cognitive impairment in stroke patients and provide more targeted guidance for clinical practice. Therefore, this study used a logistic regression model and a decision tree model to analyze the influencing factors of cognitive impairment in stroke patients in order to provide a reliable basis for clinical prevention and treatment of cognitive impairment after stroke.

### 4.4 Association between optimism and cognitive impairment in stroke patients

Spearman’s correlation analysis showed that there was a negative correlation between optimism and cognitive impairment in stroke patients, that is, the higher the optimism score, the lower the cognitive impairment score, which was consistent with the cross-sectional study (14). The results of regression analysis and structural equation modeling showed that the optimistic status of Chinese stroke patients was directly associated with the level of cognitive impairment. Theoretical models suggest that optimism is linked to positive health in indirect and direct ways. Health behavior is considered an indirect way. Optimism is associated with better health behaviors in the elderly, such as more active exercise and smoking cessation (40). Individuals with higher optimism tend to report healthier diets and more effective stress management (14, 40). Studies also suggest that more optimistic individuals may have better self-regulation ability and experience higher levels of positive emotions. More optimistic individuals tend to have more confidence in the future to deal with difficult living conditions and work harder to solve problems, but they are also more willing to adjust when the goal cannot be achieved (41). Additionally, optimistic individuals are also more likely to seek social support, which is a key environmental factor associated with cognitive preservation.

### 4.5 Relationship between coping styles and cognitive impairment in stroke patients

Palamarchuk proposed a multi-level model to explain the role of cognitive processing, decision-making, and behavior in coping with stress (42). Cognitive introspection is associated with better cognitive performance, which suggests that positive coping strategies are correlated with better mental health and cognitive ability. This is completely consistent with the results of this study. This study incorporates the variables of optimism, coping, and cognitive outcomes that were previously discussed separately into a unified theoretical framework and validates the mediation model, providing a more refined map for understanding the process by which post-stroke psychosocial factors affect cognitive health. Our results suggest that clinical evaluation should not be limited to neurological deficits alone. Routine screening of patients’ optimistic tendencies and coping strategies can help identify individuals at higher risk of cognitive decline. Targeted psychological interventions, such as positive psychology interventions to enhance optimism or cognitive-behavioral therapy to train adaptive coping skills, can be integrated into stroke rehabilitation programs as potential non-pharmacological cognitive protection strategies. Longitudinal studies are needed in the future to confirm causal directions. Meanwhile, intervention studies can examine whether enhancing optimism and adaptive coping strategies can effectively improve cognitive outcomes. In addition, exploring the relationship between biological markers and these psychological variables will provide a deeper insight into the mechanisms of the heart–brain connection.

### 4.6 Mediating role of coping style between optimism and cognitive impairment

We demonstrate that coping strategies play an important mediating role in optimism and cognitive function. Poor optimism in stroke patients may trigger negative coping strategies, related to cognitive impairment, while optimistic emotions can trigger positive coping strategies, reducing the occurrence of cognitive impairment after stroke survival. The biopsychosocial model of cognitive impairment suggests that cognition is not only determined by biological and physical health factors, but psychological and social factors can also affect cognitive health and dementia risk (43). In addition, certain personality traits are also related to cognitive health, such as an outgoing personality being associated with better cognitive function (44). Ryo’s study showed that negative avoidance coping is significantly correlated with significant cognitive decline (45), which is consistent with the results of this study. Another study (46) also suggests that coping strategies partially mediate the relationship between empathy and burnout. Optimism is a positive personality trait that is linked to cognitive function indirectly through its association with coping strategies, which is consistent with our research findings. In addition to encouraging a positive attitude, supporting the development of effective coping skills may also be an important consideration for cognitive health after stroke.

### 4.7 Clinical implications

This cross-sectional study demonstrates the mediating role of coping style in the relationship between optimism and cognitive impairment in stroke patients. From a clinical perspective, the identified mediating role of coping style suggests that psychological interventions for post-stroke patients could extend beyond general support to specifically target enhancing optimistic thinking and promoting positive coping strategies. Medical staff help patients maintain a positive attitude, and a positive coping style may help identify patients at higher risk of cognitive decline, thereby enabling timely and personalized psychological care. Furthermore, stroke recovery is a long-term process that continues at home. Patient-centered care models, such as early discharge planning and home-based rehabilitation, increasingly emphasize the role of patient self-management and adaptive psychological resources (47). Future interventions could target these modifiable factors to support survivors in the more challenging, less structured home environment. For future clinical research, our study paves the way for developing and testing targeted interventions. It provides a rationale for future randomized controlled trials to examine whether interventions such as optimism training or cognitive behavioral therapy designed to improve coping skills can effectively delay cognitive impairment in stroke survivors.

## 5 Limitations and prospects

Several limitations of this study should be noted. First and foremost, the cross-sectional design is a major limitation. The data were collected at a single time point, which prevents us from drawing causal conclusions about the relationships between optimism, coping style, and cognitive impairment. While our model was based on theoretical rationale, it is possible that the relationships are bidirectional or that unmeasured confounding variables account for the observed associations. The mediation analysis, therefore, identifies a statistically significant indirect association, not a proven causal pathway.

Second, a significant limitation is the lack of assessment for depression and anxiety. These highly prevalent conditions post-stroke are not only strong correlates of cognitive impairment but are also intricately linked to both dispositional optimism and coping styles. For instance, depression can directly diminish an optimistic outlook, promote avoidant coping, and exacerbate cognitive complaints. Therefore, it is plausible that the observed association between optimism and cognitive impairment, partially mediated by coping style, could be confounded by underlying depressive or anxious symptomatology acting as a common cause. While our model is grounded in theory, the absence of these critical covariates means we cannot rule out the possibility that they account for a portion of the shared variance among our study variables. Future studies must prioritize their inclusion as covariates or competing mediators to isolate the unique contribution of the optimism-coping pathway.

Future research should focus on two key areas. First, a prospective cohort study should be designed to assess optimism and coping strategies during acute hospitalization, with repeated evaluations of cognitive function at 3, 6, and 12 months after stroke. Second, key covariates such as depression and anxiety should be measured to distinguish their effects from those of the hypothesized optimism and coping styles.

## Data Availability

The datasets presented in this article are not publicly available because they contain sensitive information, including data that could compromise patient privacy. Requests for access should be directed to the corresponding author.
